# Dataset on the suitability of groundwater for drinking and irrigation purposes in the Sarabanga River region, Tamil Nadu, India

**DOI:** 10.1016/j.dib.2020.105255

**Published:** 2020-02-07

**Authors:** P. Balamurugan, P.S. Kumar, K. Shankar

**Affiliations:** a Department of Civil Engineering, M.Kumarasamy College of Engineering, Tamilnadu, India; b Department of Civil Engineering, University College of Engineering, Ariyalur, Tamilnadu, India; c Department of Applied Geology, School of Applied Natural Science, Adama Science and Technology University, Ethiopia

**Keywords:** Groundwater, Water quality index, Drinking purpose, Irrigation purpose

## Abstract

The present datasets reveal that to assess the suitability of groundwater quality for drinking and irrigation uses in both Pre and Post Monsoon Season in Sarabanga River region, Tamilnadu, India based on various water quality indices. A total of 50 groundwater samples were collected in different location in a research area. Water Quality Index (WQI) is a number which indicates the suitability of water for drinking purpose. Sodium Absorption Ratio (SAR), Permeability Index (PI), Residual Sodium Carbonate (RSC), Percentage Sodium (%Na), Kelly Ratio (KR) and Magnesium Hazards (MH) are index value which elaborates the fitness of groundwater for agriculture uses. The WQI value for groundwater in both seasons reveals that 74.5 sq.km and 37.24 sq.km of the area were unfit for domestic purposes. Based on irrigation indices, almost all sample locations are suitable for irrigation purposes. The dataset demonstrates how water quality indices would be applied to policymakers to manage, handle and sustainably improve society at large.

**Table:** Specifications Table

Subject	Environmental Engineering
Specific subject area	Groundwater Quality
Type of data	Tables, Figures
How data were acquired	All water samples were analyzed according to the Standard Methods using potentiometer method by digital pH meter (Instrument Model: DPH-500, Global make) for pH, digital conductivity meter (Instrument Model: DCM-900, Global make) for EC and titration method was used to determine the Total Hardness, Calcium, Magnesium and Chloride. Nitrate and Sulphate were estimated with UV Spectrophotometer.
Data format	Raw, Analyzed
Parameters for data collection	All water samples were collected in 1 L pre-cleaned high density polyethylene bottles (HDPE), transferred to the laboratory and were stored at 4 °C and analyzed within 2 days of sampling following APHA (2012) methods.
Description of data collection	All the samples were analyzed according to APHA method for physicochemical parameters viz., pH, EC, TDS, TH, Ca^2+^, Mg^2+^, Na^+^, K^+^, HCO_3_^−^, NO_3_^−^, SO_4_^2−^, Cl^−^ and F^−^.To determine the suitability of groundwater using WQI and Irrigation indices.
Data source location	Sarabanga River region, Tamilnadu, India
Data accessibility	Data are available in this article and supplementary file.
Related research article	P.S. Kumar & P. Balamurugan, Evaluation of Groundwater Quality for Irrigation Purpose in Attur Taluk, Salem, Tamil Nadu, India. Water & Energy International, 61(4) (2018), 59–64 [1].

**Table:** 

**Value of the Data** • The dataset provides information on the assessment of groundwater quality status in Sarabanga river region. • The data are considered as the most important for improvement the quality of groundwater. • The data is useful to take remedial action against carcinogenic and non-carcinogenic effect in human being. • This dataset gives a clear idea about the impact of risk in continuous consumers as well as researcher and professionals in this field.

## 1 Data description

**Fig. 1:**
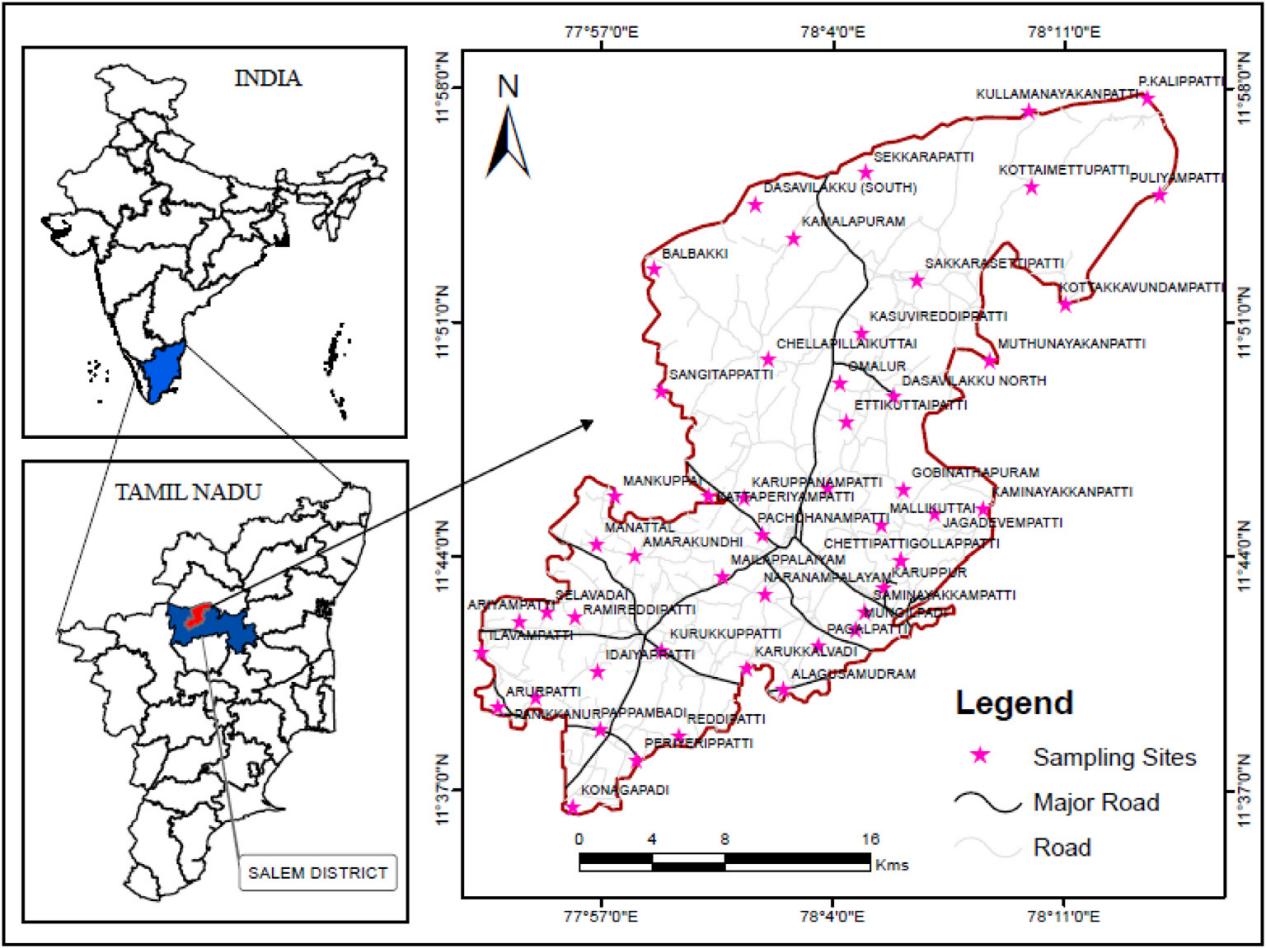
The base map and location of sampling sites.

**Fig. 2:**
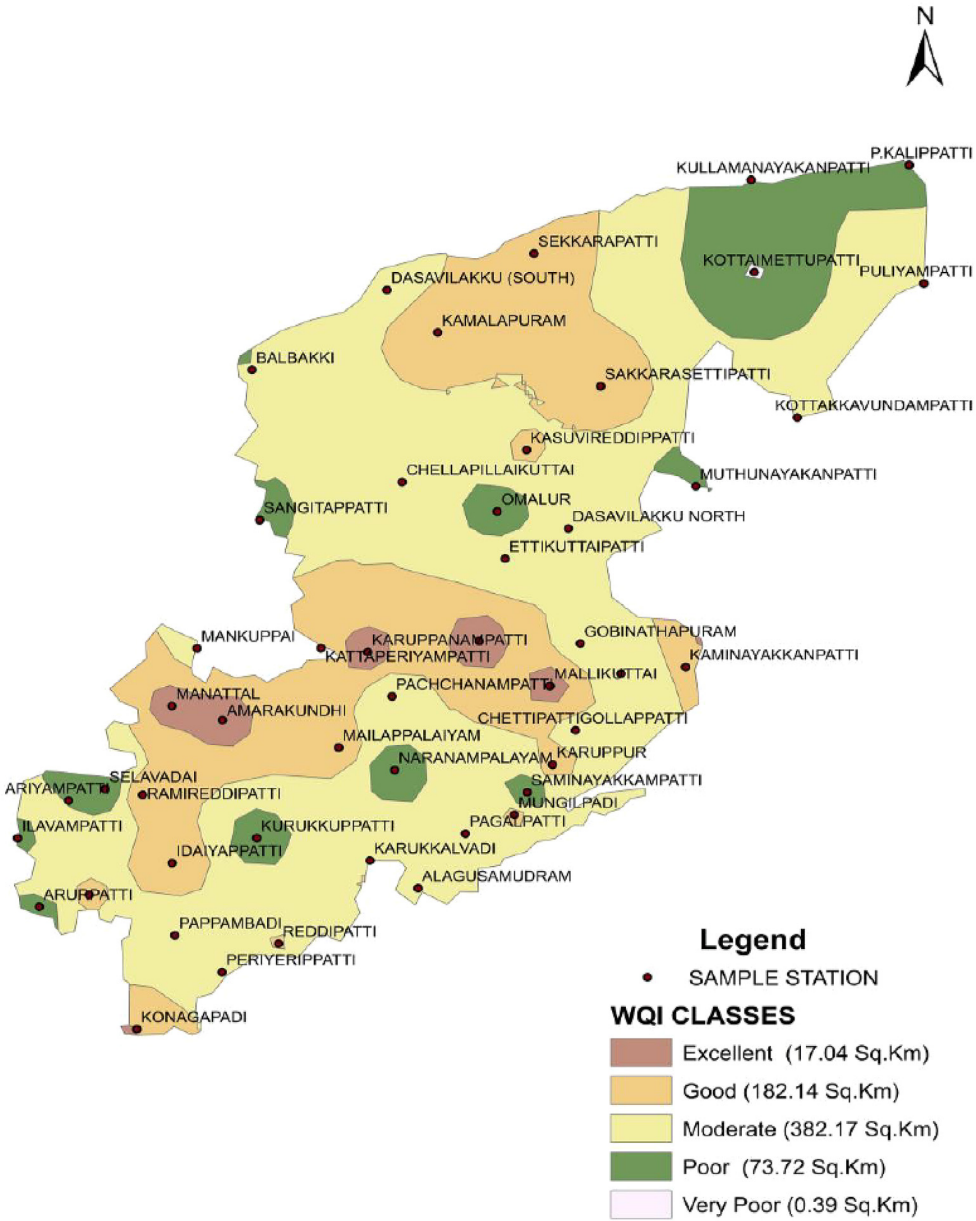
Spatial distribution of WQI in the Sarabanga River during the pre-monsoon period.

**Fig. 3:**
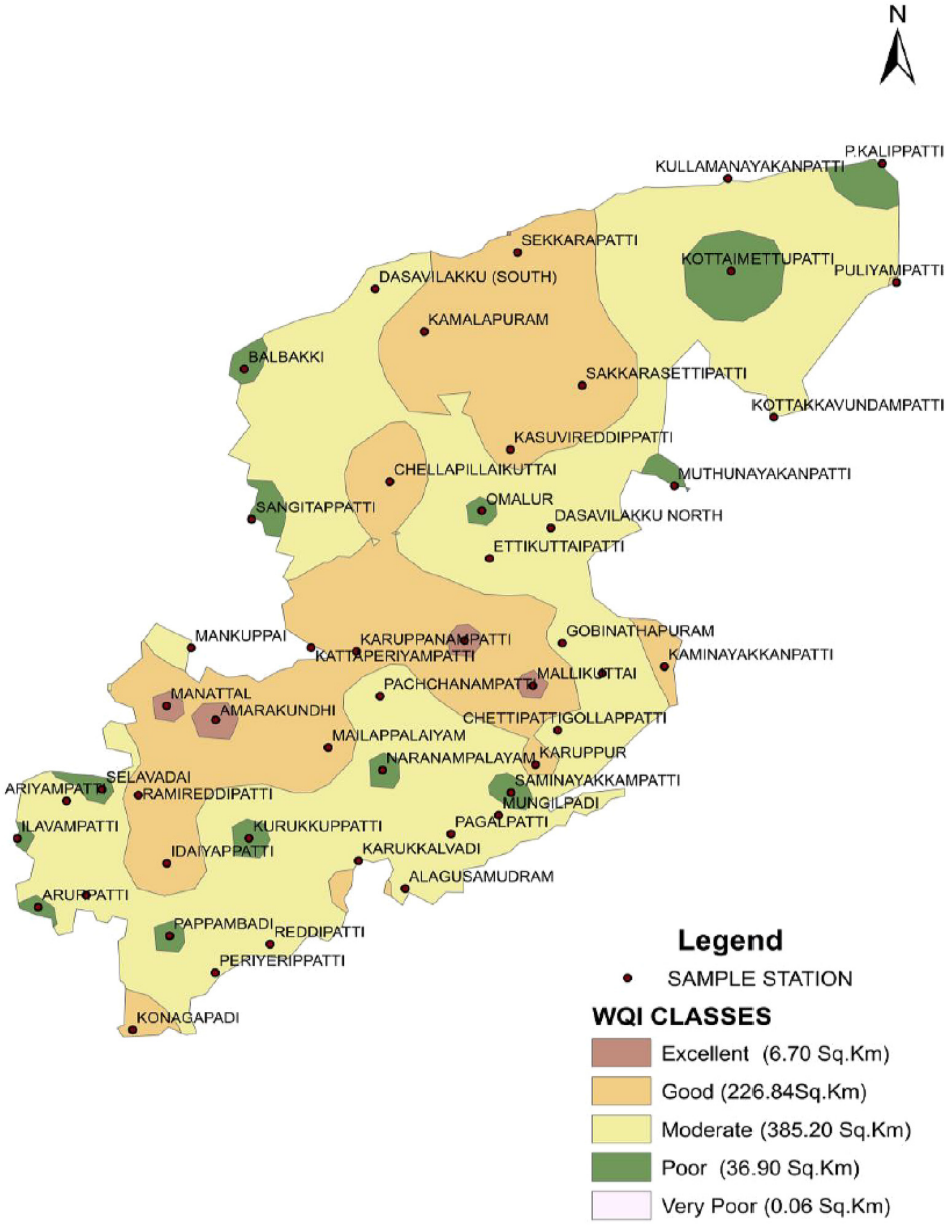
Spatial distribution of WQI in the Sarabanga River during the Post-monsoon Period.

**Fig. 4:**
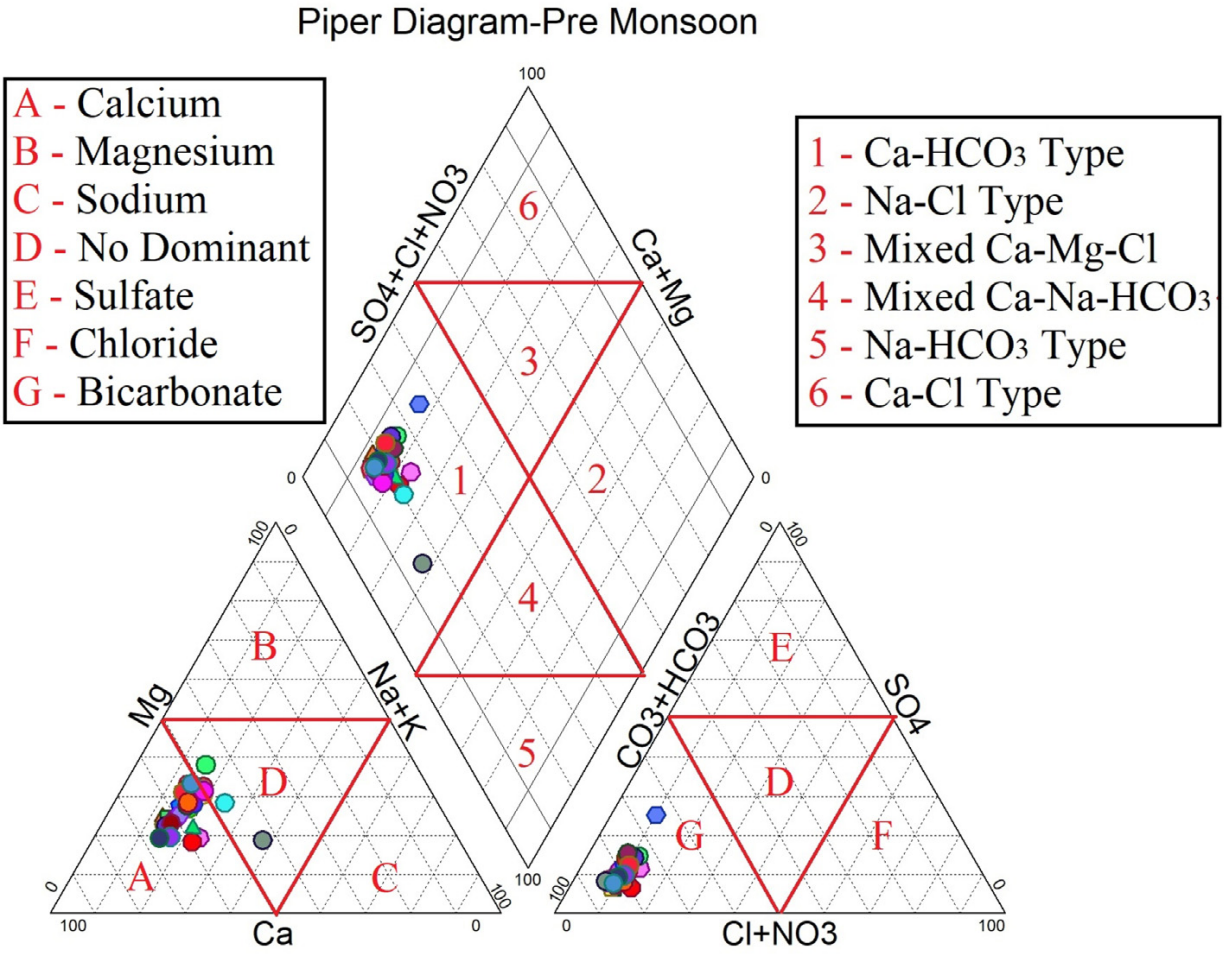
Piper diagram – Pre monsoon Period.

**Fig. 5:**
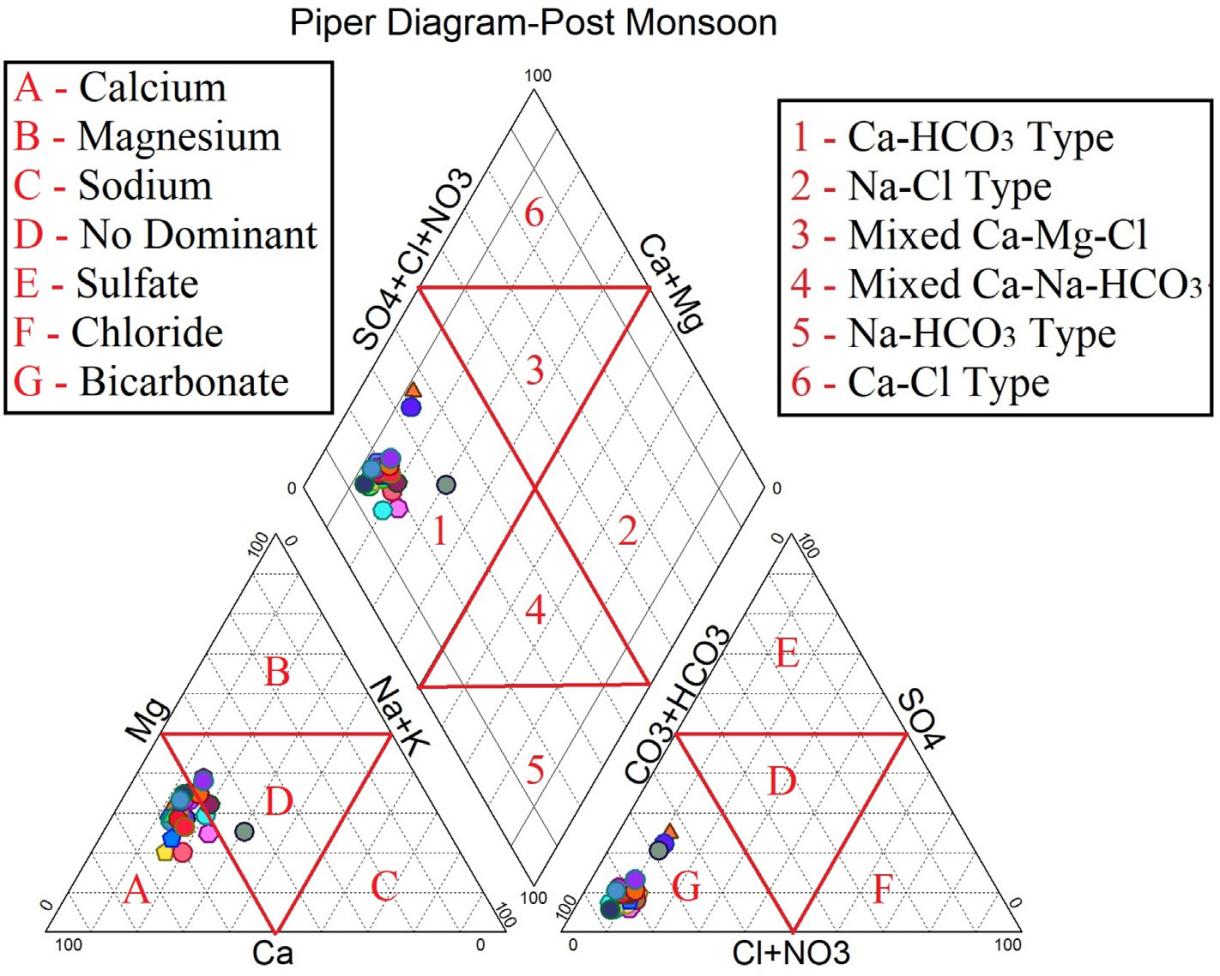
Piper diagram – Pre monsoon Period.

**Fig. 6:**
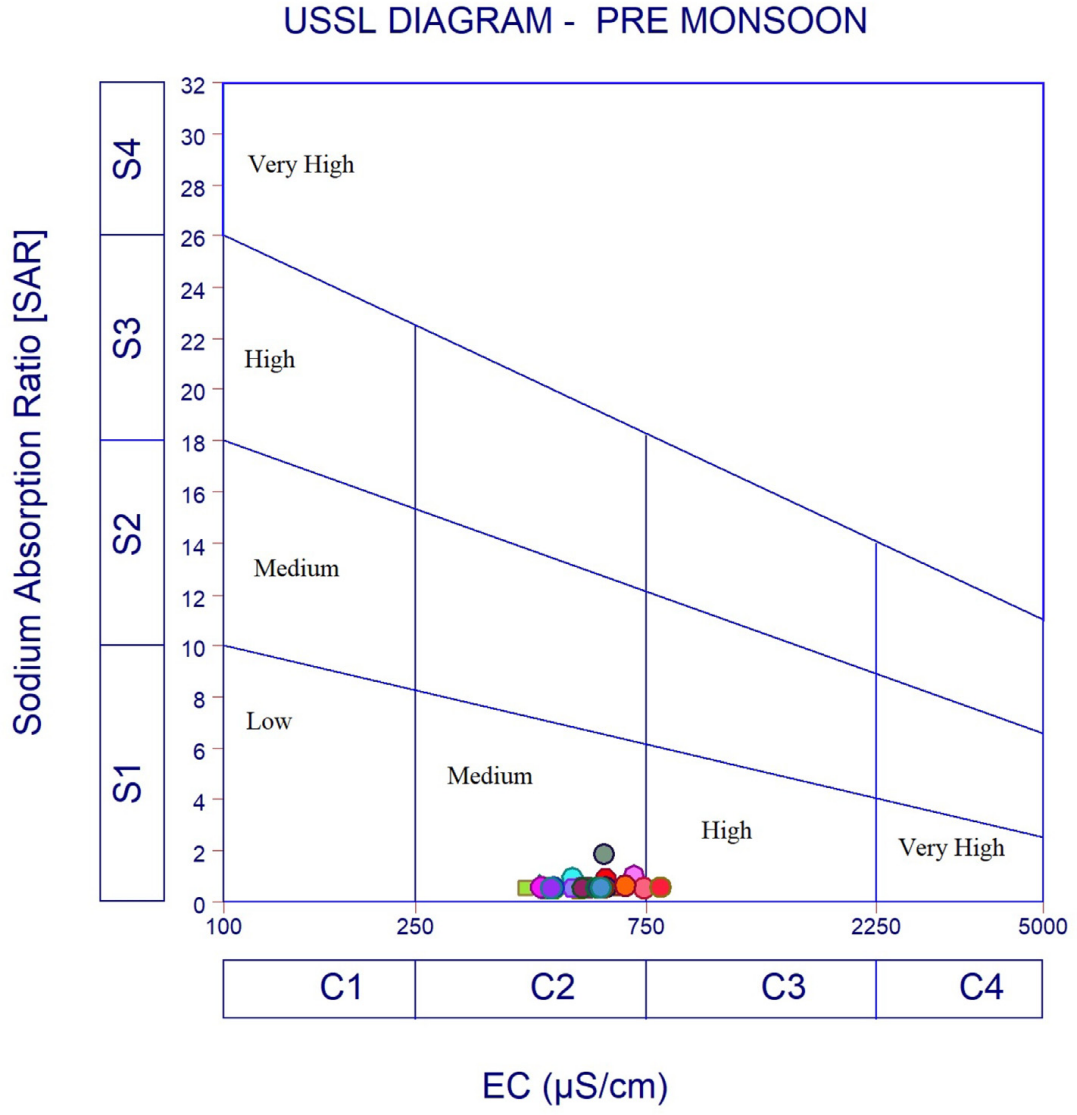
USSL Classification of groundwater during Pre-monsoon.

**Fig. 7:**
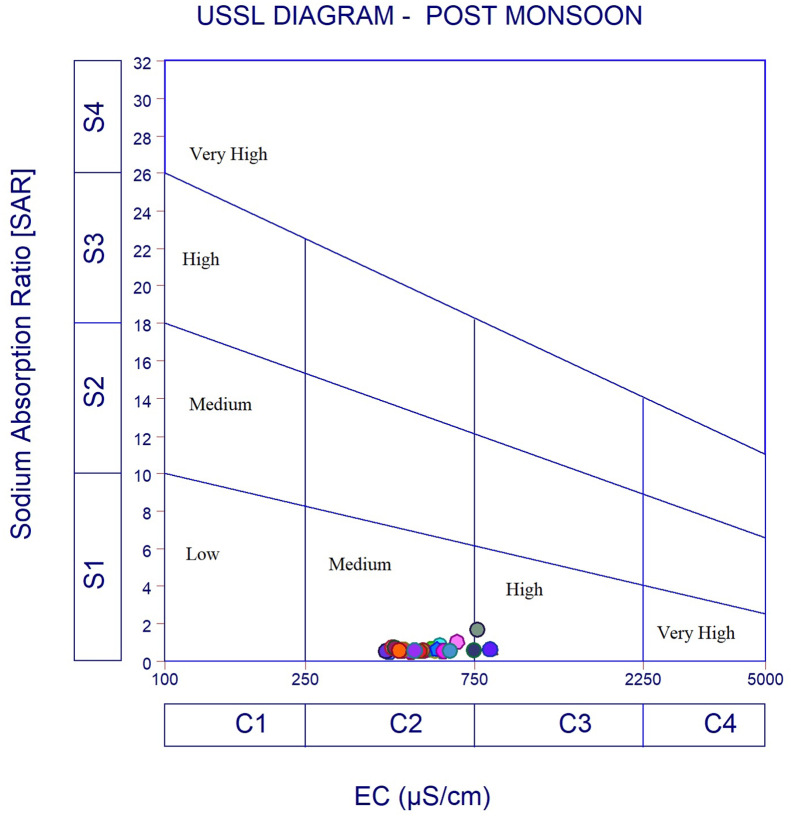
USSL Classification of groundwater during Post monsoon.

**Fig. 8:**
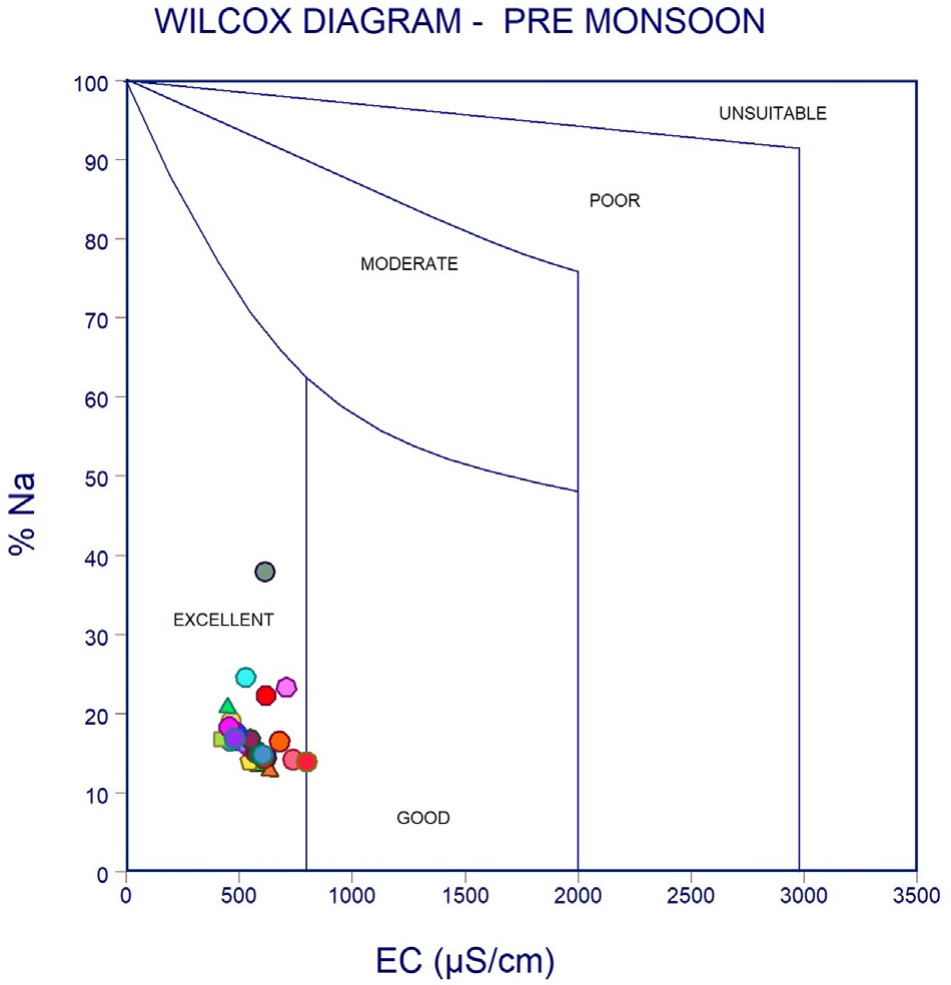
Wilcox Classification of groundwater during Pre-monsoon.

**Fig. 9:**
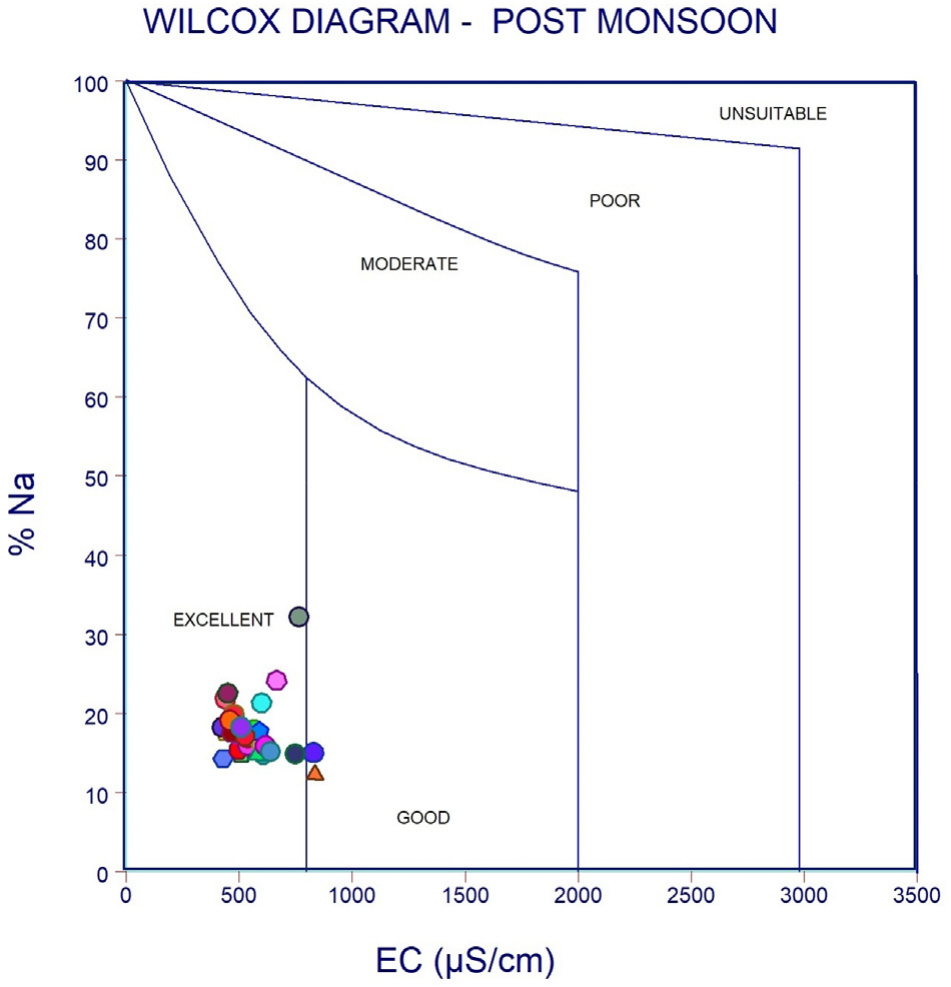
Wilcox Classification of groundwater during Post monsoon.

The dataset in this research paper reveals the hydrochemical properties of groundwater and its nature for drinking and irrigation purposes in the Sarabanga river region. A Sarabanga river flows through the Omalur taluk, Salem District in the state of Tamil Nadu, India (Fig. 1). Omalur is a well-developing taluk in the district. It is bounded with geographic coordinates of 11°73′ N and 78°07’ E at an average altitude of 298 m from the mean sea level. The average rainfall intensity is 100 mm per year. Groundwater is the only source of people for their daily needs [1]. The data presented deal with monitoring of physical and chemical characteristics of groundwater such as pH, EC, TDS, TH, Ca^2+^, Mg^2+^, Na^+^, K^+^, HCO_3_^−^, NO_3_^−^, SO_4_^2−^, Cl^−^ and F^−^. Fig. 1 shows the location and sampling points of the research area. Fig. 2, Fig. 3 show the nature of groundwater quality (WQI) in the pre- and post-monsoon period. Fig. 4, Fig. 5 describes the hydro-chemical type of groundwater in both seasons. Fig. 6, Fig. 7 reveal that, relationship between sodium absorption ratio and electrical conductivity properties in groundwater. Fig. 8, Fig. 9 describe the relationship between the percentage of sodium and electrical conductivity in groundwater. The detailed chemical analysis procedure was illustrated in Table 1. A maximum, minimum, average and standard deviation of all groundwater parameters in pre- and post-monsoon are shown in Table 2. The physicochemical parameters for the WQI calculation with the BIS standard are shown in Table 3. The computed WQI was compared to the range of WQI for drinking water [14] in order to identify the water category as shown in Table 4. To assess the suitability of groundwater for irrigation purposes in the research area using irrigation indices such as Sodium Absorption Ratio (SAR), Residual Sodium Carbonate (RSC), Permeability Index (PI), Magnesium Hazards (MH), Percentage Sodium (%Na), Kelly Ratio (KR) were calculated by the formulas presented in Table 5. All data determined groundwater concentrations used in these computations were in meq/l. Suitability, range and Class of water during the pre- and post-monsoon period have been tabulated in Table 6. An interrelationship between each parameter and statistical analysis of groundwater in both seasons are shown in Table 7, Table 8. The raw data provided in supplementary file.

**Table 1:** Standard procedures for each parameter [2].

S.No	Parameter	Units	Methods	Field kit/Instruments
1	pH		Potentiometer	pH meter, (DPH-500, Global make)
2	Electrical Conductivity	μs/cm	–	EC meter, (DCM-900, Global make)
3	Total dissolved solids	mg/L	–	TDS meter, (Aqua make)
4	Total alkalinity	mg/L	Sulfuric acid	–
5	Total hardness	mg/L	Standardized EDTA	–
6	Calcium	mg/L	Standardized EDTA	–
7	Magnesium	mg/L	Standardized EDTA	–
8	Chloride	mg/L	Standardized silver nitrate	–
9	Sulphate	mg/L	–	UV visible spectrophotometer
10	Potassium	mg/L	Flame photometric	Flame Photometer
11	Sodium	mg/L	Flame photometric	Flame Photometer

**Table 2:** Statistical summary of groundwater during Pre and Post-Monsoon Seasons.

Ions	Pre-Monsoon				Post-Monsoon				WHO 2011	BIS 1991
	Max	Min	Mean	SD	Max	Min	Mean	SD		
pH	8.3	6.8	7.5	0.3	8.5	6.7	7.5	0.4	6.5–8.5	6.5–8.5
EC	3180.0	343.0	1167.1	566.1	3215.0	326.0	1165.8	573.0	1000	400
TDS	2035.2	219.5	747.0	362.3	2057.6	208.6	746.1	366.7	500	500
TH	510.4	133.4	319.6	80.8	591.8	180.0	299.9	69.6	120	300
Ca^2+^	96.0	23.0	68.1	19.1	100.0	36.0	70.3	16.5	75	75
Mg^2+^	67.0	13.0	36.4	11.4	88.0	13.0	30.2	12.0	50	30
Na^+^	460.0	49.0	116.4	78.4	332.0	30.0	121.8	70.9	200	100
K^+^	42.0	5.0	14.0	9.3	103.0	3.0	26.4	20.1	12	10
NO_3_^−^	180.0	6.0	69.7	46.4	180.0	0.0	75.4	49.8	45	45
Cl^−^	508.0	36.0	151.8	89.2	524.0	40.0	150.1	90.1	250	250
SO_4_^2-^	713.0	23.0	131.3	135.5	1159.0	26.0	151.1	190.1	250	250
F^−^	1.6	0.0	0.8	0.5	1.5	0.1	0.8	0.4	1.5	1.5
HCO_3_^−^	966.0	44.6	308.5	149.5	927.0	15.6	288.8	152.0	120	200
SAR	11.0	1.2	2.9	2.0	9.9	0.8	3.2	2.0	–	–
MAR	69.6	29.2	46.8	8.0	122.4	42.3	81.8	19.5	–	–
%Na	79.2	25.6	43.8	13.2	79.2	19.9	47.9	12.5	–	–
KR	3.7	0.3	0.9	0.7	7.4	0.4	1.9	1.3	–	–
PI	98.9	41.7	62.3	13.7	90.3	34.1	64.2	12.2	–	–
RSC	8.6	−6.8	−1.3	2.8	10.1	−5.6	−1.3	2.8	–	–

**Table 3:** Assigned and relative weight for WQI computation with BIS standards [8,15].

Chemical parameters	BIS standards desired limit	Weight (wi)	Relative Weight (Wi)
SO_4_^2-^	200	5	0.13
NO_3_^−^	45	5	0.13
F^−^	1.5	5	0.13
Cl^−^	250	5	0.13
TDS	500	5	0.13
Na^+^	100	4	0.11
Ca^2+^	75	3	0.08
Mg^2+^	30	3	0.08
K^+^	10	2	0.05
HCO_3_^−^	200	1	0.03
		∑wi = 38	∑Wi = 1.00

**Table 4:** WQI range and classification for drinking purposes [25].

S·NO.	RANGE	WQI Classes	Pre - Monsoon		Post - Monsoon	
			No. of samples	% of samples	No. of samples	% of samples
1	0–25	Excellent	7	14	6	12
2	26–50	Good	13	26	14	28
3	51–75	Moderate	16	32	16	32
4	76–100	Poor	13	26	13	26
5	>100	Very poor	1	2	1	2

**Table 5:** Summary of water quality indices for irrigation [8,9,15].

Parameters	Formula
Sodium Absorption Ratio (SAR)	Na^+^/(Ca^2+^+Mg^2+^)/2)^½^
Residual Sodium Carbonate (RSC)	(HCO_3_^−^ + CO_3_^2−^) – (Ca^2+^+ Mg^2+^)
Permeability Index (PI)	[Na^+^+ (HCO_3_^−^)^1/2^/(Ca^2+^+Mg^2+^+Na^+^)]×100
Magnesium Hazards (MH)	[Mg^2+^/(Ca^2+^ + Mg^2+^)] × 100
Percentage Sodium (% Na)	[(Na^+^+K^+^)/(Ca^2+^+Mg^2+^+Na^+^+K^+^)]×100
Kelly Ratio (KR)	Na^+^/(Ca^2+^ + Mg^2+^)

**Table 6:** Classification of groundwater for irrigation purpose during Pre- and post-monsoon.

Parameters	Range	Water Class	Pre-monsoon		Post-monsoon	
			No. of Samples	% of samples	No. of Samples	% of samples
Sodium Absorption Ratio (SAR)	0–10	Excellent	49	98	50	100
	10–18	Good	1	2	NIL	0
	18–26	Doubtful	NIL	0	NIL	0
	>26	Unfit	NIL	0	NIL	0
Residual Sodium Carbonate (RSC)	<1.25	Good	50	100	50	100
	1.25–2.5	Doubtful	0	0	0	0
	>2.5	Unfit	0	0		0
Permeability Index (PI)	>75	Class-I	4	8	4	08
	25–75	Class-II	46	92	46	92
	<25	Class-III	NIL	0	NIL	0
Magnesium Hazards (MH)	<50	Suitable	35	70	42	84
	>50	Unsuitable	15	30	8	16
Percentage Sodium (% Na)	<20	Excellent	NIL	0	1	2
	20–40	Good	25	50	12	24
	40–60	Permissible	18	36	29	58
	60–80	Doubtful	7	14	8	16
	>80	Unfit	NIL	0	NIL	0
Kelly Ratio (KR)	<1	Suitable	37	74	33	66
	>1	Unsuitable	13	26	17	34

**Table 7:** Correlation Coefficient between parameters during Pre-Monsoon.

Ions	pH	EC	TDS	TH	Ca	Mg	Na	K	NO_3_	CL	SO_4_	F
**pH**	1.00											
**EC**	**−0.34**	1.00										
**TDS**	**−0.34**	1.00	1.00									
**TH**	0.25	**−0.09**	**−0.09**	1.00								
**Ca**	0.33	0.04	0.04	0.85	1.00							
**Mg**	0.09	**−0.20**	**−0.20**	0.85	0.45	1.00						
**Na**	**−0.22**	0.01	0.01	0.04	**−0.05**	0.12	1.00					
**K**	**−0.07**	0.00	0.00	**−0.13**	**−0.17**	−0.05	0.08	1.00				
**NO**_**3**_	**−0.15**	0.29	0.29	**−0.01**	**−0.07**	0.04	0.28	−0.05	1.00			
**CL**	**−0.26**	0.18	0.18	**−0.20**	**−0.24**	**−0.11**	**−0.15**	**−0.02**	**−0.24**	1.00		
**SO**_**4**_	**−0.13**	**−0.22**	**−0.22**	**−0.02**	**−0.13**	0.10	0.02	**−0.13**	**−0.18**	0.21	1.00	
**F**	0.27	**−0.11**	**−0.11**	0.32	0.16	0.39	**−0.14**	**−0.01**	0.10	**−0.16**	**−0.04**	1.00

**Table 8:** Correlation Coefficient between parameters during Post-Monsoon.

Ions	pH	EC	TDS	TH	Ca	Mg	Na	K	NO_3_	CL	SO_4_	F
pH	1.00											
EC	**−0.33**	1.00										
TDS	**−0.33**	1.00	1.00									
TH	**−0.10**	**−0.06**	**−0.06**	1.00								
Ca	**−0.05**	**−0.16**	**−0.16**	0.72	1.00							
Mg	**−0.10**	0.06	0.06	0.81	0.18	1.00						
Na	**−0.30**	0.19	0.19	**−0.18**	**−0.09**	**−0.19**	1.00					
K	0.42	**−0.02**	**−0.02**	0.09	0.05	0.08	**−0.21**	1.00				
NO_3_	0.08	0.26	0.26	0.04	**−0.15**	0.18	0.26	0.13	1.00			
CL	**−0.27**	0.14	0.14	**−0.19**	**−0.18**	**−0.12**	0.01	**−0.33**	**−0.14**	1.00		
SO_4_	**−0.07**	**−0.23**	**−0.23**	**−0.08**	**−0.12**	**−0.02**	**−0.04**	**−0.36**	**−0.29**	0.19	1.00	
F	0.23	**−0.16**	**−0.16**	0.05	0.04	0.03	**−0.03**	0.16	0.07	**−0.10**	**−0.08**	1.00

## 2 Experimental design, materials, and methods

In order to assess the groundwater quality for drinking and irrigation purpose, a total of 50 groundwater samples were collected from a bore well at an average depth of 120 feet in river region during the pre-monsoon and post-monsoon seasons (the year of 2017). Samples were collected in a washed and dried polythene bottles at a capacity of 1000ml. Collected samples were kept at 4 °C and it transferred to the laboratory immediately for further analysis. The hydrochemical properties of groundwater were analyzed for the concentration of hydrogen ions (pH), total dissolved solids, alkalinity, Hardness, major cation like calcium magnesium, sodium, potassium and anion concentrations like chloride, sulphate, bicarbonate using Standard procedure APHA [2]. During sample collection, handling, preservation and analysis, standard procedures recommended by the American Public Health Association [[2], [3], [4], [5], [6]] were followed to ensure data quality and consistency. The summary of the measured physicochemical parameters and the calculation of the maximum, minimum, mean and standard deviations found in different water samples and the final data of the physicochemical concentration were compared with the World Health Organization [6] and the Indian Bureau standards [7], as shown in Table 2. In the research data, various irrigation indices and ratios of groundwater such as Sodium Absorption Ratio (SAR), Residual Sodium Carbonate (RSC), Permeability Index (PI), Magnesium Hazards (MH), Percentage Sodium (%Na), Kelly Ratio (KR) were also identified as shown in Table .5 [8,9]. The US Salinity Laboratory diagram [10] is widely used for the evaluation of irrigation waters where SAR is plotted against EC (Fig. 6, Fig. 7) and demonstrates that groundwater samples fall into categories C2S1 and C3S1, indicating medium to high salinity and low sodium type for both seasons. Wilcox diagram [11] is used to determine the classification and viability of groundwater for irrigation purposes based on sodium percent and EC (Fig. 8, Fig. 9) and shows that groundwater samples are excellent to good for both seasons. Based on all irrigation indices data from revels that the groundwater quality in the Sarabanga river region is good in post-monsoon and few sample locations are affected by higher concentration calcium and magnesium ions due to lithology and rock water interactions. Statistical analysis was carried out using the Statistical Package for Social Sciences (SPSS 10.0) [12]. The correlation coefficient values among the parameters for groundwater are presented in Table 7, Table 8 In order to describe groundwater quality and also possible pathways of geochemical changes, major ion chemical data have been drawn on the Piper Trilinear diagram [13] in Fig. 4, Fig. 5. Data were made available in a format that is accessible via GIS (ArcGIS -Spatial Analyst tool) [15]. Inverse distance weighted (IDW) interpolation method was used to produce spatial variation maps for determined Water quality index map in groundwater of research area.

### 2.1 Water Quality Index calculation for drinking

The Water Quality Index (WQI) assessed the suitability of groundwater for drinking purposes and compared the values of different water quality parameters with those of the World Health Organization [6] and the Indian Bureau standard [7] guidelines [8,15]. In order to calculate the WQI, the weights for the physical and chemical parameters were determined with respect to the relative importance of the overall quality of the water for drinking water purposes [8]. The following steps are involved in WQI computing:

1. The maximum weight assigned is five and the minimum is one. The highest w_i_ was assigned to parameters that has a significant health effect [15]. F^−^ was assigned the highest w_i_ followed by SO_4_^2−^, NO_3_^−^, Ca^2+^, Cl^−^, TDS, Mg^2+^, Na^+^, and K^+^ as shown in Table 3. The least weight is assigned for HCO_3_^−^. Each parameter has been assessed according to relevance in drinking quality of groundwater (Table 3) [8,15].
2. The relative weights (W_i_) is computed by the following equation (1):

(1) $${\text{W}}_{\text{i}}\;=\;{\text{w}}_{\text{i}}/\sum_{i=1}^{n}{\text{w}}_{\text{I}}$$

Where, Wi = Relative weight, wi = Weight of each parameter, n = number of parameters.

3. Quality rating (Eq. (2)),

(2) $${\text{q}}_{\text{i}}\;=\;\left(\text{Ci /Si}\right)\text{×}100$$

Where, q_i_= Quality rating for i_th_ parameter, Ci= Concentration of i_th_ parameter in groundwater sample, and Si= desirable limit set by BIS.

4. Sub-index (Eq. (3)),

(3) $${\text{SI}}_{\text{i}}\;=\;{\text{W}}_{\text{i}}\;\text{×}\;{\text{q}}_{\text{i}}$$

5. Water quality index (Eq. (4)),

(4) $$\text{WQI}\;=\;\sum {\text{SI}}_{\text{i}}$$

WQI range suggested by Ref. [14] was used to identify the groundwater type (Table 4). The spatial map shows that the overall water quality in the area was excellent, good water, moderate water, poor water and very poor water in Figs. 2 and 3. However, in both seasons, the overall quality of groundwater for drinking purposes is moderate to poor.
