# Supplementary material for: Dataset on the suitability of groundwater for drinking and irrigation purposes in the Sarabanga River region, Tamil Nadu, India
Source: Data Brief. 2020 Feb 7;29:105255. doi: 10.1016/j.dib.2020.105255 (PMC7031326; doi:10.1016/j.dib.2020.105255)
Supplement: Multimedia component 2 [file mmc2.pdf]

Table 1

Hydrochemical parameters of groundwater samples during Pre-monsoon in Sarabanga river region

S.NO	VILLAGES	Physical Parameter				Chemical Parameter								
		pH	EC	TDS	TH	Ca^{2+}	Mg^{2+}	Na^{+}	K^{+}	HCO_3^-	NO_3^-	Cl^-	SO_4^{2-}	F^-
			µs/cm	mg/L										
1	Alagusamudram	7.31	1300	832	210	46	23	134	18	34.41	180	130	290	0.5
2	Amarakundhi	7.23	1267	811	133	32	13	224	16	117.69	84	108	113	0
3	Anaikavundanpatti	7.13	1150	736	176	36	21	54	19	138.30	12	196	147	0
4	Ariyampatti	7.45	1410	902	266	49	35	136	26	123.09	6	182	33	1.5
5	Arurpatti	7.45	1564	1001	383	86	41	116	5	14.81	65	200	713	1.3
6	Balbakki	7.11	1220	781	189	23	32	196	16	128.74	126	193	233	1.2
7	Chellapillaikuttai	7.37	1445	925	353	59	50	110	10	93.53	42	104	198	1
8	Chettipatti	7.21	1595	1021	314	63	38	86	9	39.73	57	142	167	1
9	Dasavilakku (south)	7.2	1110	710	510	94	67	88	18	117.11	116	163	160	1
10	Dasavilakku north	7.46	1755	1123	397	85	45	84	32	19.63	59	154	113	1
11	Ettikuttaipatti	7.24	1260	806	429	96	46	108	12	54.11	14	168	214	1
12	Gobinathapuram	7.26	1490	954	278	52	36	220	5	53.89	165	56	34	0.9
13	Gollappatti	7.55	1520	973	399	79	49	124	28	167.43	123	60	67	0.7
14	Idaiyappatti	7.25	1670	1069	363	86	36	150	22	49.48	43	36	78	0.3
15	Ilavampatti	7.12	1870	1197	340	80	34	118	6	64.18	135	148	89	1.2
16	Jagadevempatti	6.86	1760	1126	329	56	46	208	13	182.21	178	169	68	1
17	Kamalapuram	7.29	1500	960	310	65	36	52	12	187.13	12	144	86	0.4
18	Kaminayakkanpatti	7.33	1350	864	332	59	45	460	22	137.87	138	122	191	0
19	Karukkalvadi	7.34	1235	790	382	77	46	134	5	44.82	69	102	62	1.1
20	Karuppanampatti	7.16	1830	1171	305	86	22	74	13	128.42	48	280	47	0
21	Karuppur	7.01	3180	2035	222	46	26	102	12	136.65	64	508	32	0.6
22	Kasuvireddippatti	7.5	437	280	139	26	18	112	42	88.60	57	200	155	0.7
23	Kattaperiyampatti	6.82	343	220	176	36	21	90	10	44.41	19	288	363	0.5
24	Konagapadi	7.5	519	332	291	67	30	62	8	118.83	20	168	552	0.2
25	Kottaimettupatti	7.94	453	290	422	70	60	68	9	119.63	70	112	351	1.5

Table 1 (continued)

S.NO	VILLAGES	Physical Parameter				Chemical Parameter								
		pH	EC	TDS	TH	Ca^{2+}	Mg^{2+}	Na^{+}	K^{+}	HCO_3^{-}	NO_3^{-}	Cl^{-}	SO_4^{2-}	F^{-}
			µs/cm			mg/L								
26	Kottakkavundampatti	8	564	361	337	61	45	50	8	139.46	18	448	246	0.8
27	Kullamanayakanpatti	7.98	489	313	413	78	53	110	10	59.04	23	67	164	1.2
28	Kurukkuppatti	7.43	1640	1050	277	63	29	64	22	157.57	17	142	183	1.6
29	Mailappalaiyam	7.49	1850	1184	383	94	36	138	11	158.07	75	282	50	0.4
30	Mallikuttai	7.26	489	313	247	56	26	68	9	146.42	19	197	171	0
31	Manattal	7.42	536	343	347	73	40	72	10	49.90	33	184	149	0
32	Mankuppai	7.25	615	394	335	60	45	112	20	104.67	66	164	150	0.9
33	Mungilpadi	7.43	485	310	437	96	48	390	5	93.83	14	130	182	0.6
34	Muthunayakanpatti	7.85	406	260	388	96	36	194	40	34.81	41	84	41	1.5
35	Naranampalayam	7.45	437	280	365	62	51	75	13	142.00	51	143	41	1.6
36	Omalur	7.66	429	275	436	84	55	110	7	153.24	59	130	33	1.5
37	P.Kalippatti	7.5	483	309	297	63	34	162	11	141.00	106	89	33	1.3
38	Pachchanampatti	8.01	444	284	393	90	41	63	11	63.25	96	167	43	1
39	Pagalpatti	7.79	494	316	298	60	36	68	8	123.25	41	172	121	0.8
40	Panikkanur	7.43	1550	992	366	92	33	96	7	145.35	156	123	31	0.5
41	Pappambadi	7.67	1265	810	238	49	28	59	6	136.48	132	46	58	1
42	Periyerippatti	7.78	1320	845	296	84	21	100	5	143.78	59	44	56	1
43	Puliyampatti	7.26	1555	995	394	92	40	56	11	151.09	96	56	25	0.7
44	Ramireddipatti	7.14	1330	851	242	59	23	108	5	158.39	59	105	26	0.4
45	Reddipatti	8	1410	902	317	76	31	68	13	165.70	39	87	23	0.6
46	Sakkarasettipatti	8.33	1205	771	319	80	29	95	6	173.00	48	88	29	0.2
47	Saminayakkampatti	8.33	1180	755	295	72	28	86	6	180.31	96	100	32	1.5
48	Sangitappatti	8.03	1363	872	293	81	22	49	11	187.61	82	95	29	1.3
49	Sekkarapatti	7.56	1280	819	294	60	35	66	40	194.92	66	124	45	0.2
50	Selavadai	7.4	1305	835	325	69	37	49	19	202.22	91	188	49	1.6

Table 2

Hydrochemical parameters of groundwater samples during Post-monsoon in Sarabanga river region

S.NO	VILLAGES	Physical Parameter				Chemical Parameter								
		pH	EC	TDS	TH	Ca^{2+}	Mg^{2+}	Na^{+}	K^{+}	HCO_3^-	NO_3^-	Cl^-	SO_4^{2-}	F^-
			μs/cm	mg/L										
1	Alagusamudram	7.28	1340	858	260	48	34	96	16	28.95	140	154	304	0.3
2	Amarakundhi	7.12	1568	1004	242	59	23	328	26	116.45	90	111	108	0.1
3	Anaikavundanpatti	7.24	1160	742	257	52	31	30	28	135.95	0	188	151	0.2
4	Ariyampatti	7.3	1380	883	290	57	36	156	33	118.83	10	180	314	1.5
5	Arurpatti	7.32	1589	1017	310	63	37	172	9	11.56	60	189	672	1.3
6	Balbakki	7.23	1147	734	299	57	38	252	19	124.21	150	197	231	1.2
7	Chellapillaikuttai	7.42	1480	947	371	81	41	114	18	91.18	50	114	203	0.5
8	Chettipatti	6.98	1610	1030	346	89	30	70	12	35.47	50	138	170	0.6
9	Dasavilakku (south)	7.14	1113	712	592	92	88	54	23	113.85	130	165	155	0.9
10	Dasavilakku north	7.4	1785	1142	396	86	44	60	36	14.39	52	142	118	0.7
11	Ettikuttaipatti	7.28	1280	819	358	84	36	236	13	49.85	3	168	249	1
12	Gobinathapuram	7.56	1540	986	349	87	32	172	5	49.33	180	52	34	0.6
13	Gollappatti	7.64	1495	957	321	76	32	72	32	162.09	100	64	70	0.7
14	Idaiyappatti	7.2	1660	1062	254	59	26	90	29	43.84	40	40	85	0.3
15	Ilavampatti	7.22	1850	1184	274	57	32	92	13	59.64	130	128	76	1.2
16	Jagadevempatti	6.92	1740	1114	180	49	14	304	19	178.65	170	165	62	1
17	Kamalapuram	7.51	1505	963	199	55	15	136	9	181.89	20	144	65	0.6
18	Kaminayakkanpatti	7.47	1343	860	264	63	26	332	16	132.61	150	120	193	0.3
19	Karukkalvadi	7.25	1240	794	341	84	32	148	3	39.93	56	100	63	1
20	Karuppanampatti	7.16	1840	1178	296	61	35	104	16	122.18	56	287	43	0.4
21	Karuppur	7.02	3215	2058	209	49	21	136	20	132.08	76	524	31	0.6
22	Kasuvireddippatti	7.37	424	271	215	63	14	99	26	83.24	49	198	196	0.7
23	Kattaperiyampatti	6.71	326	209	283	87	16	83	11	40.22	12	290	391	0.5
24	Konagapadi	7.83	505	323	225	49	25	56	8	113.51	34	176	1159	0.2
25	Kottaimettupatti	7.89	432	276	272	86	14	54	6	113.41	11	120	342	1.5

Table 2 (continued)

S.NO	VILLAGES	Physical Parameter				Chemical Parameter								
		pH	EC	TDS	TH	Ca^{2+}	Mg^{2+}	Na^{+}	K^{+}	HCO_3^-	NO_3^-	Cl^-	SO_4^{2-}	F^-
			μs/cm	mg/L										
26	Kottakkavundampatti	8.1	543	348	251	74	16	114	8	132.30	87	456	269	0.8
27	Kullamanayakanpatti	7.56	492	315	343	96	25	192	24	53.90	6	59	168	0.9
28	Kurukkuppatti	7.47	1630	1043	324	72	35	56	18	152.25	12	128	183	1.4
29	Mailappalaiyam	7.13	1800	1152	321	99	18	228	9	153.92	63	200	52	0.4
30	Mallikuttai	7.25	465	298	310	63	37	99	10	140.18	24	203	179	0.1
31	Manattal	7.49	506	324	363	81	39	109	11	44.76	41	184	154	0.1
32	Mankuppai	7.37	589	377	321	76	32	110	28	99.49	64	165	153	1
33	Mungilpadi	7.85	414	265	342	89	29	56	12	88.59	11	126	189	0.8
34	Muthunayakanpatti	7.7	424	271	264	76	18	156	14	29.32	49	92	41	1.5
35	Naranampalayam	7.53	490	314	386	92	38	110	65	136.76	66	139	42	1.3
36	Omalur	7.83	442	283	211	63	13	122	45	147.98	64	134	35	1.3
37	P.Kalippatti	7.65	500	320	313	61	39	156	58	133.75	131	91	33	1.3
38	Pachchanampatti	8.06	469	300	344	85	32	66	43	56.12	103	164	42	1
39	Pagalpatti	7.98	509	326	276	48	38	91	35	115.59	84	176	44	0.8
40	Panikkanur	7.73	1560	998	410	100	39	109	24	137.16	180	112	29	0.5
41	Pappambadi	7.88	1355	867	272	56	32	68	27	127.75	166	50	95	1.1
42	Periyerippatti	8.04	1263	808	316	69	35	152	52	134.53	95	46	91	1
43	Puliyampatti	7.27	1565	1002	308	92	19	73	84	141.30	109	52	29	0.7
44	Ramireddipatti	7.21	1310	838	330	73	36	129	24	148.08	66	73	26	0.4
45	Reddipatti	8.23	1340	858	359	81	38	59	103	154.85	56	85	28	0.6
46	Sakkarasettipatti	8.49	1260	806	218	56	19	86	43	161.63	68	88	27	0.2
47	Saminayakkampatti	8.36	1207	772	205	36	28	94	48	168.40	109	100	31	1.5
48	Sangitappatti	8.09	1323	847	307	85	23	53	49	175.18	96	95	27	1.3
49	Sekkarapatti	7.44	1245	797	229	49	26	83	26	181.95	85	127	55	0.2
50	Selavadai	7.34	1020	653	270	52	34	71	16	188.73	115	204	48	1.3

Table 3

Quality of groundwater in Sarabanga river region for irrigation purpose during Pre and Post monsoon

S.NO	Villages	Pre-Monsoon (meq/L)						Post Monsoon (meq/L)					
		SAR	%NA	RSC	MH	PI	KR	SAR	%NA	RSC	MH	PI	KR
1	Alagusamudram	4.03	60.03	-3.61	45.19	65.69	1.39	2.59	46.89	-4.61	53.88	52.59	0.80
2	Amarakundhi	8.44	79.20	-0.66	40.12	89.71	3.65	9.17	75.53	-2.83	39.13	81.95	2.95
3	Anaikavundanpatti	1.77	44.58	-1.20	49.03	65.63	0.67	0.81	28.21	-2.82	49.58	43.57	0.25
4	Ariyampatti	3.63	55.27	-3.25	54.09	65.26	1.11	3.98	56.79	-3.73	51.02	65.17	1.17
5	Arurpatti	2.58	40.30	-7.42	44.02	43.57	0.66	4.25	55.48	-5.94	49.20	58.33	1.21
6	Balbakki	6.20	70.27	-1.63	69.65	81.08	2.25	6.34	65.72	-3.82	52.37	73.32	1.84
7	Chellapillaikuttai	2.55	41.66	-5.48	58.29	50.86	0.68	2.58	42.22	-5.84	45.50	50.08	0.67
8	Chettipatti	2.11	38.77	-5.61	49.87	45.43	0.60	1.64	32.67	-6.25	35.73	38.70	0.44
9	Dasavilakku (south)	1.69	29.59	-8.19	54.03	37.16	0.38	0.97	19.89	-9.82	61.20	26.33	0.20
10	Dasavilakku north	1.83	36.02	-7.61	46.61	36.39	0.46	1.31	30.86	-7.58	45.76	30.20	0.33
11	Ettikuttaipatti	2.27	36.85	-7.66	44.14	42.49	0.55	5.43	59.70	-6.24	41.41	64.34	1.43
12	Gobinathapuram	5.74	63.57	-4.64	53.31	69.48	1.72	4.01	52.18	-6.06	37.76	58.26	1.07
13	Gollappatti	2.70	43.38	-5.15	50.57	52.74	0.68	1.75	38.08	-3.60	40.98	50.11	0.49
14	Idaiyappatti	3.43	49.42	-6.43	40.84	53.89	0.90	2.46	47.81	-4.26	42.09	53.52	0.77
15	Ilavampatti	2.79	43.77	-5.71	41.21	51.65	0.76	2.42	44.18	-4.40	48.08	53.04	0.73
16	Jagadevempatti	4.99	58.77	-3.50	57.53	68.96	1.38	9.86	79.22	-0.52	32.03	88.89	3.68
17	Kamalapuram	1.28	29.28	-3.05	47.74	47.40	0.36	4.19	60.70	-0.82	31.03	77.49	1.49
18	Kaminayakkanpatti	10.98	75.58	-4.32	55.71	80.70	3.01	8.89	73.76	-2.95	40.50	80.84	2.73
19	Karukkalvadi	2.98	43.85	-6.86	49.63	49.69	0.76	3.49	48.84	-6.06	38.59	55.00	0.94
20	Karuppanampatti	1.84	36.79	-3.95	29.67	50.10	0.53	2.63	45.44	-3.77	48.62	57.19	0.76
21	Karuppur	2.98	51.68	-2.09	48.25	66.88	1.00	4.10	60.63	-1.83	41.41	73.47	1.42
22	Kasuvireddippatti	4.13	68.15	-1.28	53.31	79.44	1.75	2.94	53.65	-2.80	26.82	64.07	1.00
23	Kattaperiyampatti	2.95	54.20	-2.78	49.03	64.09	1.11	2.15	40.75	-4.91	23.27	48.16	0.64
24	Konagapadi	1.58	33.30	-3.83	42.48	48.10	0.46	1.62	36.97	-2.52	45.70	55.23	0.54
25	Kottaimettupatti	1.44	27.44	-6.46	58.57	38.27	0.35	1.42	31.49	-3.47	21.17	48.12	0.43

Table 3 (continued)

S.NO	Villages	Pre-Monsoon (meq/L)						Post Monsoon (meq/L)					
		SAR	%NA	RSC	MH	PI	KR	SAR	%NA	RSC	MH	PI	KR
26	Kottakkavundampatti	1.18	26.07	-4.44	54.89	41.33	0.32	3.13	50.76	-2.71	26.29	64.92	0.99
27	Kullamanayakanpatti	2.36	37.92	-7.26	52.85	44.24	0.58	4.51	56.70	-5.85	30.05	61.42	1.22
28	Kurukkuppatti	1.67	37.70	-2.87	43.16	52.82	0.50	1.35	30.92	-3.81	44.50	45.39	0.38
29	Mailappalaiyam	3.07	45.09	-5.00	38.71	55.75	0.78	5.54	61.25	-3.77	23.07	70.55	1.54
30	Mallikuttai	1.88	39.25	-2.42	43.37	57.11	0.60	2.45	42.44	-3.67	49.20	55.80	0.70
31	Manattal	1.68	32.82	-6.11	47.47	40.10	0.45	2.49	40.92	-6.43	44.26	47.08	0.65
32	Mankuppai	2.66	44.56	-4.97	55.30	53.44	0.73	2.67	46.13	-4.70	40.98	54.37	0.74
33	Mungilpadi	8.12	66.17	-7.17	45.19	70.82	1.94	1.32	28.66	-5.25	34.96	39.69	0.36
34	Muthunayakanpatti	4.29	54.97	-7.18	38.21	56.78	1.09	4.18	57.53	-4.70	28.09	62.53	1.29
35	Naranampalayam	1.71	33.03	-4.92	57.57	45.37	0.45	2.44	45.52	-5.35	40.52	50.48	0.62
36	Omalur	2.29	36.28	-6.10	51.92	47.18	0.55	3.66	60.52	-1.59	25.39	72.39	1.26
37	P.Kalippatti	4.09	55.22	-3.48	47.09	65.96	1.19	3.84	56.94	-3.79	51.33	63.70	1.09
38	Pachchanampatti	1.38	27.76	-6.75	42.90	35.44	0.35	1.55	36.62	-5.76	38.31	39.91	0.42
39	Pagalpatti	1.71	34.68	-3.89	49.74	49.13	0.50	2.38	46.78	-3.46	56.63	56.75	0.72
40	Panikkanur	2.18	37.35	-4.85	37.17	49.81	0.57	2.34	39.51	-5.74	39.14	48.57	0.58
41	Pappambadi	1.67	36.42	-2.39	48.52	55.53	0.54	1.80	40.20	-3.07	48.52	53.11	0.54
42	Periyerippatti	2.53	43.07	-3.42	29.19	57.31	0.73	3.72	55.68	-3.83	45.55	62.99	1.05
43	Puliyampatti	1.23	25.64	-5.30	41.76	38.86	0.31	1.81	46.39	-3.57	25.41	50.91	0.52
44	Ramireddipatti	3.02	49.94	-2.10	39.13	66.17	0.97	3.09	48.52	-3.87	44.85	59.12	0.85
45	Reddipatti	1.66	34.16	-3.51	40.22	49.52	0.47	1.36	42.06	-4.33	43.62	43.29	0.36
46	Sakkarasettipatti	2.31	40.19	-3.46	37.42	55.34	0.65	2.53	52.63	-1.44	35.88	66.99	0.86
47	Saminayakkampatti	2.18	39.77	-2.83	39.08	56.66	0.63	2.86	56.46	-1.03	56.19	70.92	1.00
48	Sangitappatti	1.25	29.19	-2.69	30.94	48.67	0.36	1.32	36.72	-2.97	30.86	48.10	0.38
49	Sekkarapatti	1.68	39.87	-2.60	49.03	53.27	0.49	2.38	48.26	-1.31	46.67	65.87	0.79
50	Selavadai	1.18	28.75	-3.11	46.93	45.86	0.33	1.88	39.34	-2.01	51.89	57.89	0.57

Table 4

Calculation of WQI for individual water samples

S.NO.	Villages	Pre - Monsoon		Post - Monsoon	
		WQI	Type of water	WQI	Type of water
1	Alagusamudram	43.87	Good	32.70	Good
2	Amarakundhi	10.12	Excellent	16.63	Excellent
3	Anaikavundanpatti	6.48	Excellent	18.71	Excellent
4	Ariyampatti	87.67	Poor	87.37	Poor
5	Arurpatti	83.80	Poor	81.18	Poor
6	Balbakki	75.41	Poor	79.44	Poor
7	Chellapillaikuttai	65.85	Moderate	40.57	Good
8	Chettipatti	63.81	Moderate	40.46	Good
9	Dasavilakku (south)	71.54	Moderate	69.43	Moderate
10	Dasavilakku north	67.60	Moderate	51.22	Moderate
11	Ettikuttaipatti	63.52	Moderate	61.71	Moderate
12	Gobinathapuram	64.32	Moderate	52.86	Moderate
13	Gollappatti	56.78	Moderate	53.96	Moderate
14	Idaiyappatti	27.55	Good	24.98	Excellent
15	Ilavampatti	77.46	Poor	77.21	Poor
16	Jagadevempatti	68.34	Moderate	63.98	Moderate
17	Kamalapuram	30.93	Good	40.52	Good
18	Kaminayakkanpatti	19.17	Excellent	34.01	Good
19	Karukkalvadi	71.80	Moderate	63.44	Moderate
20	Karuppanampatti	10.27	Excellent	32.31	Good
21	Karuppur	40.86	Good	41.08	Good
22	Kasuvireddippatti	47.31	Good	46.09	Good
23	Kattaperiyampatti	29.95	Good	29.15	Good
24	Konagapadi	22.92	Excellent	26.73	Good
25	Kottaimettupatti	99.72	Poor	90.17	Poor

Table 4 (continued)

S.NO.	Villages	Pre - Monsoon		Post - Monsoon	
		WQI	Type of water	WQI	Type of water
26	Kottakkavundampatti	59.79	Moderate	61.11	Moderate
27	Kullamanayakanpatti	80.92	Poor	57.54	Moderate
28	Kurukkuppatti	93.03	Poor	83.87	Poor
29	Mailappalaiyam	37.29	Good	30.92	Good
30	Mallikuttai	9.03	Excellent	15.98	Excellent
31	Manattal	13.48	Excellent	19.74	Excellent
32	Mankuppai	60.15	Moderate	64.88	Moderate
33	Mungilpadi	44.71	Good	55.83	Moderate
34	Muthunayakanpatti	94.05	Poor	90.30	Poor
35	Naranampalayam	97.43	Poor	82.66	Poor
36	Omalur	95.68	Poor	81.21	Poor
37	P.Kalippatti	83.33	Poor	86.73	Poor
38	Pachchanampatti	73.76	Moderate	73.29	Moderate
39	Pagalpatti	57.22	Moderate	61.23	Moderate
40	Panikkanur	45.59	Good	50.62	Moderate
41	Pappambadi	70.08	Moderate	79.82	Poor
42	Periyerippatti	66.84	Moderate	72.78	Moderate
43	Puliyampatti	51.67	Moderate	49.71	Good
44	Ramireddipatti	30.14	Good	33.17	Good
45	Reddipatti	48.43	Good	52.51	Moderate
46	Sakkarasettipatti	31.26	Good	31.96	Good
47	Saminayakkampatti	100.11	Very Poor	100.27	Very Poor
48	Sangitappatti	85.84	Poor	87.39	Poor
49	Sekkarapatti	25.78	Good	24.34	Excellent
50	Selavadai	97.90	Poor	82.54	Poor
